# Dental Amalgam Fillings and Multiple Sclerosis: A Nationwide Population-Based Case–Control Study in Taiwan

**DOI:** 10.3390/ijerph17082637

**Published:** 2020-04-12

**Authors:** Chien-Fang Tseng, Kun-Huang Chen, Hui-Chieh Yu, Fu-Mei Huang, Yu-Chao Chang

**Affiliations:** 1 School of Dentistry, Chung Shan Medical University, Taichung 40201, Taiwan; chenfang8858@gmail.com (C.-F.T.); yujessica7@gmail.com (H.-C.Y.); 2 Department of Artificial Intelligence, CTBC Business School, Tainan 709, Taiwan; khchen9@gmail.com; 3 Department of Dentistry, Chung Shan Medical University Hospital, Taichung 40201, Taiwan; hfm0118@csmu.edu.tw

**Keywords:** multiple sclerosis, dental amalgam fillings, nationwide population, case–control study, mercury

## Abstract

Multiple sclerosis (MS) is an inflammatory neurological disease characterized by autoimmune-mediated demyelination of the central nervous system. Genetic and environmental factors may contribute to the development of MS. This has not been confirmed yet. Dental amalgam has long been controversial in MS due to its mercury content but the toxicological implications of mercury-containing amalgam fillings (AMF) for MS remain to be elucidated. We conducted a case–control study to investigate the association between AMF and the risk of MS from the Taiwanese National Health Insurance Research Database (NHIRD). Case (*n* = 612) and control (*n* = 612) groups were matched by sex, age, urbanization level, monthly income, and Charlson comorbidity index by propensity score matched with a 1:1 ratio from 2000 to 2013. Differences between cases and controls was not statistically significant (OR: 0.82, 95% CI = 0.65–1.05). In subjects stratified by gender, MS was also not associated with AMF for women (OR: 0.743, 95% CI = 0.552–1.000) and men (OR: 1.006, 95% CI = 0.670–1.509), respectively. In summary, this Taiwanese nationwide population-based case–control study did not find an association between MS and AMF.

## 1. Introduction

Multiple sclerosis (MS) is an immune disorder of the central nervous system. The abnormal inflammation of the brain and spinal cord causes damage to axonal myelin sheath, which leads to multiple neurological abnormalities [1]. Patients with MS may experience any neurological symptoms, with autonomic, visual, motor, and sensory deficits most often. The exact symptoms vary depending on the actual location of the patient’s lesion [2]. Several environmental exposures such as Epstein–Barr virus, low levels of Vitamin D, and smoking has been associated with an increased risk of developing MS [3].

Previous reports have suggested that people with MS may be more likely to identify a higher prevalence of dental caries [4,5,6,7]. Mercury vapor from dental amalgam preparation and restoration could underlie the relation with MS. Dental amalgam is constituted of approximately 50% mercury and copper, tin, silver, and zinc to form solid amalgams, and has been used continuously in dentistry for nearly 200 years. It is known that amalgam fillings (AMF) could continually emit mercury vapor, which is increased by drinking hot fluids, chewing, eating, and brushing [8].

Recently, a case–control reported a relationship between AMF and MS [9]. However, in previous cross-sectional studies, there were no significant relationship between AMF and MS [10,11,12]. Therefore, the association between MS and AMF needs further investigation. In this study, a population-based case–control study was conducted to investigate this putative association using the Taiwan National Health Insurance Research database (NHIRD).

## 2. Materials and Methods

### 2.1. Database and Study Design

The Longitudinal Health Insurance Database 2010 (LHID2010) includes all the original claims data and registration files from 2000 to 2013 for 1 million individuals randomly sampled from the Registry for Beneficiaries of the National Health Institute (NHI) program in 2010 [13]. LHID2010′s validity has been proven by its previous use in epidemiologic and clinical research in Taiwan [14,15,16,17]. This study was approved by the Chung Shan Medical University Hospital Ethics Review Board (CSMUH No. CS2-17086).

### 2.2. Selection of Case and Control Groups

**Figure 1:**
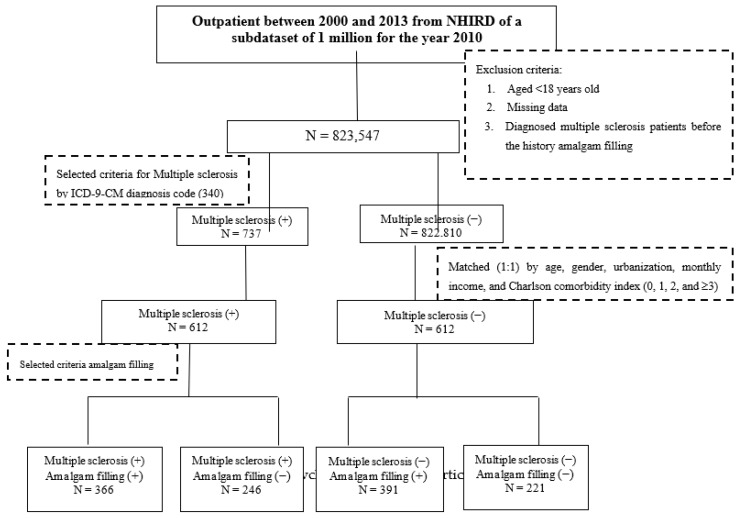
Procedures used for selection of cases from the Longitudinal Health Insurance Database 2010.

Cases from the NHI program with missing data and in individuals aged less than 18 years old were excluded. The disease diagnoses were defined according to the International Classification of Diseases, Ninth Revision, Clinical Modification (ICD-9-CM). Patients diagnosed with MS (ICD-9-CM 340) before a history of AMF were not included. Moreover, we only captured those subjects who had at least two consensus diagnoses of MS in order to increase the validity of the diagnoses. For each case, we conditionally selected comparison subjects from the general population matched by gender, age, urbanization level, socioeconomic status, and Charlson comorbidity index (CCI) using the propensity score method at a 1:1 ratio. The CCI score was indicated as 0, 1, 2, and ≥3, with higher scores indicating greater comorbidity [18]. The flowchart of the study is shown in Figure 1.

### 2.3. Exposure Assessment

The validity of the AMF sourced from LHID2010 was confirmed by the routine verification of treatment codes of the NHI system for health insurance reimbursement eligibility. We identified AMF cases by treatment codes 89001C, 89002C, 89003C, 89101C, 89102C, and 89103C of the NHI system. Potentially confounding factors, including gender, age, socioeconomic factors, urbanization, and CCI, were identified and categorized.

### 2.4. Statistical Analysis

Data analyses were performed using SPSS version 18 (SPSS, Chicago, IL, USA). Statistical analyses were performed using Student’s *t*-test for continuous variables and the chi-squared test for categorical variables. The odds ratio (OR) between case and control groups was analyzed by the chi-squared test. A multivariate logistic regression model was performed for subgroup analysis. All results are presented as ORs and 95% confidence intervals (CIs). Adjustments were made for age, gender, income, region, and CCI. Statistical significance was set at *p* < 0.05.

## 3. Results

As shown in Table 1, a final sample of 612 cases and 612 control patients were included in this study. The individuals had an average age of 50.6 ± 16.8 years. In addition, 804 (65.7%) cases were female and 420 (34.3%) were male. Demographic characteristics, including age, gender, urbanization, region, monthly income, and CCI score, were not significantly different between the cases and control groups.

As shown in Table 2, the difference between cases and controls was not statistically significant (adjusted OR: 0.823, 95% CI = 0.648–1.046).

MS was not associated with AMF regardless of gender (Table 3 and Table 4). The adjusted OR of AMF for MS was 0.743 (95% CI = 0.552–1.000) and 1.006 (95% CI = 0.670–1.509) for non-AMF for women and men, respectively.

## 4. Discussion

This is the first report to investigate the association between AMF and MS in southeast Asia. AMF was not associated with MS in this study. Our findings are in agreement with earlier studies evaluating the associations between AMF and MS in Canada [11], Italy [12], and Romania [13]. With the nationwide population derived from the NHIRD, this longitudinal sampling dataset from 2000 to 2013 made the study more representative than most, with this case–control study having more strength than previous cross-sectional designs.

A cross sectional case–control study in Iran demonstrated that levels of exposure to AMF were significantly higher in 132 individuals affected by MS than in normal persons [10]. However, this study had limited sample sizes making it difficult to estimate the AMF in patients with MS.

Many types of dental amalgams contain mercury, which can be emitted as mercury vapor [19]. In vitro studies have demonstrated that mercury can induce oxidative stress, stimulate autoimmunity, and damage DNA [20,21]. Astrocytes has been postulated in a primary role of MS [22]. Recently, inorganic mercury was found in astrocytes from autopsy-obtained tissue from a man who injected himself intravenously with metallic mercury [23]. This provides the direct evidence that mercury could play a role in the pathogenesis of MS.

Occupational exposure to elemental mercury vapor in a dental setting is primarily through inhalation during the preparation, insertion, polishing, and removal of amalgam fillings, including storage of amalgam waste before disposal [24]. Occupational elemental mercury exposure in dentists could be responsible for the higher on average prevalence of MS in dentists than that in the general population in the USA [25]. Mercury vapor levels in ambient air could exceed the maximum acceptable value during the removal of amalgam in artificial teeth when no water spray or suction is used [26]. However, it is still without any exposure assessment of mercury vapor from amalgam restorations in vivo.

Some limitations should be noted in the current study. (1) First, in Taiwan, patients with MS can be issued a catastrophic illness card to reduce their financial burden, so patients with minor manifestations of MS might not have applied for a catastrophic illness certificate. The MS diagnosis based on ICD codes might therefore affect the diagnostic accuracy for MS. However, patients suspected of having MS are referred to a board-certificated neurologist after the diagnosis is verified. (2) Second, information about the degree of disability, smoking, daily food consumption, the brands of amalgam, and the amalgam formulation was not available in the NHIRD. This may result in compromised findings and inadequate adjustment for confounding factors. (3) Third, due to this is a registry-based study, dental amalgam could already be restored in individuals’ teeth before 2000. Other metal restorations in dentition, such as inlays or crowns, were also not obtained from the NHIRD. The possible synergistic toxicity of mercury could not be excluded.

## 5. Conclusions

This nationwide population-based case–control study revealed no association between MS and AMF in Taiwan. However, since dental amalgam remains a source of mercury exposure, other alternatives for dental restorations are suggested.

## Figures and Tables

**Table 1:** Demographic characteristics of individuals in this study.

	Total (*n* = 1224)		Multiple Sclerosis (*n* = 612)		Non-Multiple Sclerosis (*n* = 612)		*p*-Value
	Population	%	Population	%	Population	%	
**Amalgam filling**	61.85%	61.85%	61.85%	61.85%	61.85%	61.85%	1.000
**Age**	50.61 ± 16.83		50.61 ± 16.46		50.69 ± 17.20		0.869
**Age groups**							0.892
18–29	129	10.54%	61	9.97%	68	11.11%	
30–39	234	19.12%	115	18.79%	119	19.44%	
40–49	225	18.38%	123	20.10%	102	16.67%	
50–59	270	22.06%	132	21.57%	138	22.55%	
60–69	176	14.38%	90	14.71%	86	14.05%	
70–79	116	9.48%	56	9.15%	60	9.80%	
>80	74	6.05%	35	5.72%	39	6.37%	
**Gender**							0.279
Female	804	65.69%	393	64.22%	411	67.16%	
Male	420	34.31%	219	35.78%	201	32.84%	
**Urbanization**							0.866
Urban	764	62.42%	389	63.56%	375	61.27%	
Suburban	334	27.29%	151	24.67%	183	29.90%	
Rural	126	10.29%	72	11.76%	54	8.82%	
**CCI**							0.376
0	267	21.81%	121	19.77%	146	23.86%	
1	287	23.45%	140	22.88%	147	24.02%	
2	204	16.67%	109	17.81%	95	15.52%	
≥3	466	38.07%	242	39.54%	224	36.60%	
**Monthly income**							0.579
<NT$ 20,000	954	77.94%	472	77.12%	482	78.76%	
NT$ 20,000–40,000	166	13.56%	87	14.22%	79	12.91%	
>NT$ 40,000	104	8.50%	53	8.66%	51	8.33%	

**Table 2:** Odds ratio for amalgam fillings of those with a diagnosis of multiple sclerosis.

	With MS (*n* = 612)		Without MS (*n* = 612)	
	No. of Patients	%	No. of Patients	%
Non-AMF	246	40.20%	221	36.11%
AMF	366	59.80%	391	63.89%
OR (95% CI)	0.841 (0.668–1.059)		1.00	
Adjusted OR (95% CI)	0.823 (0.648–1.046)		1.00	

Abbreviations: MS—multiple sclerosis; AMF—amalgam filling; OR—odds ratio; CI—confidence interval. Adjustment by age, gender, urbanization, CCI, and monthly income.

**Table 3:** Odds ratio for females with a diagnosis of multiple sclerosis with amalgam fillings.

	Female (*n* = 804)			
	With MS (*n* = 393)		Without MS (*n* = 411)	
	No. of Patients	%	No. of Patients	%
Non-AMF	153	38.93%	135	32.85%
AMF	240	61.07%	276	67.15%
OR (95% CI)	0.767 (0.575–1.024)		1.00	
Adjusted OR (95% CI)	0.743 (0.552–1.000)		1.00	

Abbreviations: MS—multiple sclerosis; AMF—amalgam filling; OR—odds ratio; CI—confidence interval. Adjustment by age, urbanization, CCI, and monthly income.

**Table 4:** Odds ratio for males with a diagnosis of multiple sclerosis with amalgam fillings.

	Male (*n* = 420)			
	With MS (*n* = 219)		Without MS (*n* = 201)	
	No. of Patients	%	No. of Patients	%
Non-AMF	93	42.47%	86	42.79%
AMF	126	57.53%	115	57.21%
OR (95% CI)	1.013 (0.688–1.492)		1.00	
Adjusted OR (95% CI)	1.006 (0.670–1.509)		1.00	

Abbreviations: MS—multiple sclerosis; AMF—amalgam filling; OR—odds ratio; CI—confidence interval. Adjustment by age, urbanization, CCI, and monthly income.
